# Potentiating Antibody-Dependent Cellular Cytotoxicity in Triple-Negative Breast Cancer via the Humanized Anti-CD147 Antibody

**DOI:** 10.3390/antib14020036

**Published:** 2025-04-11

**Authors:** Kanyarat Thongheang, Thanathat Pamonsupornwichit, Kanokporn Sornsuwan, On-anong Juntit, Tawan Chokepaichitkool, Weeraya Thongkum, Umpa Yasamut, Chatchai Tayapiwatana

**Affiliations:** 1 Division of Clinical Immunology, Department of Medical Technology, Faculty of Associated Medical Sciences, Chiang Mai University, Chiang Mai 50200, Thailand; kanyarat_thongheang@cmu.ac.th; 2 Center of Biomolecular Therapy and Diagnostic, Faculty of Associated Medical Sciences, Chiang Mai University, Chiang Mai 50200, Thailand; thanathat.pamon@cmu.ac.th (T.P.); kanokporn.sornsuwan@cmu.ac.th (K.S.); onanong.j@cmu.ac.th (O.-a.J.); weeraya.t@cmu.ac.th (W.T.); 3 Office of Research Administration, Chiang Mai University, Chiang Mai 50200, Thailand; 4 Thailand Excellence Center for Tissue Engineering and Stem Cells, Department of Biochemistry, Faculty of Medicine, Chiang Mai University, Chiang Mai 50200, Thailand; Tawan.choke@cmu.ac.th; 5 Center of Innovative Immunodiagnostic Development, Department of Medical Technology, Faculty of Associated Medical Sciences, Chiang Mai University, Chiang Mai 50200, Thailand

**Keywords:** triple-negative breast cancer, CD147, antibody therapy, ADCC, humanized antibody

## Abstract

Background: Triple-negative breast cancer (TNBC) is an aggressive subtype with high metastatic potential, poor prognosis, and the absence of estrogen receptors, progesterone receptors, and human epidermal growth factor receptor 2 (HER2). The lack of these receptors limits the standard treatments, such as hormone therapies and HER2-targeted antibodies like trastuzumab. These challenges highlight the critical need for novel therapeutic strategies. CD147, a transmembrane glycoprotein overexpressed in TNBC, promotes tumor progression, metastasis, and chemoresistance, making it a promising therapeutic target. This study evaluates the antibody-dependent cellular cytotoxicity (ADCC) of HuM6-1B9, a humanized anti-CD147 antibody, against MDA-MB-231 cells, a TNBC model. Methods: CFSE-labelled MDA-MB-231 cells were co-cultured with PBMCs as effector cells (E:T ratio 80:1) in the presence of HuM6-1B9 and incubated for 4 h. Cells were then collected and stained with PI, and CFSE+/PI+ dead target cells were analyzed by flow cytometry. Results: Co-culturing MDA-MB-231 cells with peripheral blood mononuclear cells (PBMCs) in the presence of HuM6-1B9 demonstrated effective ADCC induction without direct cytotoxicity. HuM6-1B9 induced 54.01% cancer cell death via ADCC, significantly outperforming trastuzumab (26.14%) while sparing PBMCs. Conclusion: These findings support HuM6-1B9 as a prospective TNBC therapeutic and warrant further investigation into its clinical potential.

## 1. Introduction

Breast cancer is the most common malignancy among women worldwide, accounting for a significant proportion of cancer-related mortality [1,2]. It is a highly heterogeneous disease that is classified into subtypes based on the expression of estrogen receptors (ERs), progesterone receptors (PRs), and human epidermal growth factor receptor 2 (HER2). Among these subtypes, triple-negative breast cancer (TNBC), characterized by the absence of ER, PR, and HER2 amplification, is the most aggressive form [3,4]. TNBC constitutes approximately 10–15% of all breast cancer cases and is associated with poor prognosis and worse overall survival (OS) compared to other subtypes [5,6,7]. Standard treatment modalities, including surgery, radiotherapy, and chemotherapy—such as taxanes, anthracyclines, and poly (ADP-ribose) polymerase (PARP) inhibitors approved by the Food and Drug Administration (FDA)—often fail to achieve long-term remission due to TNBC’s high metastatic potential and tendency for chemotherapy resistance, leading to recurrence [8,9]. Furthermore, TNBC exhibits resistance to endocrine treatments and HER2-targeted antibody therapies, such as trastuzumab, due to the absence of the target molecules for these treatments, underscoring the urgent need to identify novel therapeutic targets.

Among various tumor-associated antigens (TAAs), CD147 has attention as a candidate for antibody-based therapy in TNBC [10]. CD147, also known as basigin or EMMPRIN, is a single-pass transmembrane glycoprotein belonging to the immunoglobulin superfamily and is expressed on both hematopoietic and non-hematopoietic cells [11]. It plays critical roles in physiological and pathological processes, primarily through dimerization, which activates various intracellular signaling pathways. In breast cancer, CD147 is frequently overexpressed and significantly contributes to tumor progression by promoting the production of matrix metalloproteinase 9 (MMP-9), which degrades the extracellular matrix, and vascular endothelial growth factor (VEGF), which supports angiogenesis [12,13]. Additionally, CD147 interacts with adhesion molecules such as integrins, further facilitating cancer cell adhesion, invasion, and metastasis [14,15]. Furthermore, it mediates chemoresistance through the ABC transporter G2 (ABCG2) in breast cancer, while, in hepatocellular carcinoma (HCC), it contributes to resistance via activation of the MAPK signaling pathways [16,17]. In HER2-positive cancer cells with high CD147 expression, CD147 has been associated with reduced trastuzumab efficacy, suggesting that CD147 enhances resistance to anti-HER2 antibody therapy [18]. These mechanisms underline its role in tumor growth and spread. Furthermore, CD147 expression in breast cancer is strongly linked to treatment resistance and poor prognosis, making it a compelling therapeutic target for antibody-based therapies [16,19,20,21].

Several antibodies targeting CD147 have been developed and investigated for their therapeutic potential. For instance, the murine antibody HAb18IgG has been shown to sensitize pancreatic cancer cells to chemoradiotherapy by inhibiting CD44s-pSTAT3 signaling [22]. Metuzumab, a chimeric antibody, and I131-metuximab, a murine anti-CD147 antibody labeled with radioactive iodine (I131), have demonstrated potential in esophageal cancer and HCC, respectively, by inhibiting tumor growth and metastasis [23,24]. More recently, the humanized antibody HuM6-1B9 exhibited potent complement-dependent cytotoxicity (CDC) and antibody-dependent cellular phagocytosis (ADCP) activity in acute lymphoblastic leukemia (ALL) [25,26]. DS-1471a, a humanized IgG4 monoclonal antibody targeting CD147 exerted antitumor efficacy in HCC xenograft mouse models [27]. A humanized IgG4 antibody, h4#147D, demonstrated antitumor efficacy by inducing stress response signals (e.g., JNK, p38MAPK), SMAD4 expression, and caspase-3 activation in cancer models such as pancreatic ductal adenocarcinoma (PDAC), HCC, and chronic myeloid leukemia (CML) [28]. These findings collectively highlight CD147 as a potential target for antibody-based cancer therapies.

ADCC is a vital strategy for eliminating cancer cells. This process exploits the cytotoxic activity of innate immune cells, particularly natural killer (NK) cells, to induce cancer cell death. For instance, the chimeric antibody cHAb18, which targets CD147, has been shown to promote ADCC in HCC cell lines, including SMMC-7721 and Huh-7 cells. Given the overexpression of CD147 in TNBC and its functional role in tumor progression, targeting CD147 with an antibody capable of enhancing ADCC presents an intriguing therapeutic strategy. However, the role of antibodies targeting CD147 in mediating ADCC in breast cancer, particularly TNBC, remains underexplored. This study evaluates the functional role of a humanized anti-CD147 antibody (HuM6-1B9), modified from the murine M6-1B9, which recognizes the unique ^31^EDLGS^35^ epitope of domain one on CD147 [29], in promoting ADCC against MDA-MB-231 cells, a TNBC model.

Our study provides robust preclinical evidence that HuM6-1B9 significantly enhances ADCC against TNBC cells, positioning it as a promising alternative therapeutic strategy for this aggressive malignancy. HuM6-1B9 demonstrates superior cytotoxic activity compared to trastuzumab while selectively sparing normal immune cells from off-target toxicity, underscoring its potential for clinical translation. These findings highlight the therapeutic potential of HuM6-1B9 as a precision-targeted agent and lay the groundwork for exploring combination regimens with existing immunotherapies to enhance treatment outcomes for TNBC patients.

## 2. Methods

### 2.1. Cell Lines

Stable HuM6-1B9-expressing HEK293T cells were cultured in complete DMEM, consisting of Dulbecco’s Modified Eagle Medium, 10% heat-inactivated fetal bovine serum (HI-FBS), 100 U/mL of penicillin, 100 µg/mL of streptomycin, and 2 mM of L-glutamine, which supplemented with 400 µg/mL of hygromycin B. The MDA-MB-231 breast cancer cell line was obtained from ATCC (Manassas, VA, USA) and cultured in complete DMEM at 37 °C in a humidified atmosphere with 5% CO_2_.

### 2.2. Isolation of PBMCs

PBMCs were isolated from the peripheral blood of healthy donors using Ficoll-Hypaque density gradient centrifugation at a density of 1.077 g/mL. This study was conducted under the approval of the Ethics Committee of the Faculty of Associated Medical Sciences, Chiang Mai University, Thailand (Study code AMSEC-67EX-092). Briefly, 20 mL of heparinized blood was diluted 1:1 with sterile PBS before being overlaid onto Lymphoprep™ density gradient medium (STEMCELL Technologies, Col, Vancouver, BC, Germany) in a sterile 50 mL conical tube. The tube was centrifuged at 400× *g* for 30 min at room temperature with the break off. After centrifugation, the PBMCs were harvested and resuspended with complete RPMI, consisting of Roswell Park Memorial Institute 1640 medium, 10% HI-FBS, 100 U/mL of penicillin, 100 µg/mL of streptomycin, and 2 mM of L-glutamine. Viability and cell number were assessed using the trypan blue exclusion method with Countess 3 Automated Cell Counter (Invitrogen, Thermo Fisher Scientific Inc., Waltham, MA, USA).

### 2.3. Production and Purification of HuM6-1B9

Stable HuM6-1B9-expressing HEK293T cells were cultured and sequentially adapted to 100% of serum-free medium, CDM4HEK293^TM^ (Cytiva, MXG, Wilmington, DE, USA), and supplemented with 4 mM of L-glutamine and 400 µg/mL of hygromycin B. A total of 2 × 10^8^ viable cells were seeded into the cell compartment of CELLine™1000 bioreactor flasks (Wheaton Science Products, Millville, NJ, USA) at 37 °C with 5% CO_2_ for 7 days. At the end of the culture period, the cells were harvested and centrifuged at 479× *g* to remove cell debris. The culture supernatant was collected and stored at −40 °C. After harvesting, fresh HuM6-1B9-expressing HEK293T cells and medium were added into the flask. The cycle of seeding and harvesting was repeated every 7 days. The supernatants were then thawed, pooled, and filtered through a 0.2 µm filter to remove any unwanted particles prior to the purification step. The HuM6-1B9 in the culture supernatant was purified using Protein G affinity chromatography on an ÄKTA Pure protein purification system (Cytiva, MXG, Wilmington, DE, USA). The concentration of purified HuM6-1B9 was measured using an ALLSHENG Nano-400A Micro-spectrophotometer (Hangzhou Allsheng Instruments, Hangzhou, China).

### 2.4. Determination of Purity of HuM6-1B9

Antibody purity was assessed using SDS-PAGE. Purified HuM6-1B9 (2 μg/lane) was prepared with reducing and non-reducing agent, heated at 95 °C for 10 min before loading in 12% polyacrylamide gels. The protein bands were visualized by staining with COOMASSIE^nano^ Protein Staining Solution (BIO-HELIX, New Taipei City, Taiwan, China).

In addition, Western blot analysis was conducted to confirm the presence of human immunoglobulin G (IgG). Proteins were transferred to nitrocellulose membranes and probed with horseradish peroxidase (HRP)-conjugated rabbit anti-human IgG (H + L) antibody (1:3000 dilution). The band of protein was enhanced using chemiluminescent substrate and detected by Biorad ChemiDoc^TM^ Imaging System instrument (Bio-Rad, Hercules, CA, USA).

### 2.5. Examination of Binding Activity of HuM6-1B9

The binding activity of HuM6-1B9 was evaluated by indirect ELISA. Indirect ELISA was described in a previous study [25]. Briefly, the 96-well plates were coated with 10 μg/mL of human CD147-BCCP overnight at 4 °C in a moist chamber. The following steps were performed at room temperature: the wells were washed three times with washing buffer (0.05% Tween-20 in PBS) and then blocked with 2% bovine serum albumin (BSA) in 0.05% Tween-20 in PBS for 1 h at room temperature. After washing, HuM6-1B9 (1 μg/mL) was added and incubated for 1 h. The wells were then washed and incubated with HRP-conjugated rabbit anti-human IgG antibody (1:3000 dilution) for 1 h. 3,3′,5,5′-Tetramethylbenzidine (TMB) substrate was added to develop the reaction, which was then stopped with 1 N HCl. Absorbance was measured at 450 nm using an ELISA reader (Hercuvan Lab Systems, Brixton, LDN, UK).

### 2.6. Analysis of Cell Surface Molecule on Triple-Negative Breast Cancer Cells

CD147 and HER2 expression on MDA-MB-231 cells were assessed using flow cytometry. Cells were harvested and blocked with 10% HI-FBS. Then, 10 μg/mL of HuM6-1B9 or humanized anti-HER2 antibody, trastuzumab (MedChemExpress, Monmouth Junction, NJ, USA) was added and incubated for 30 minutes on ice. After washing 3 times, the cells were stained with phycoerythrin (PE)-conjugated goat anti-human IgM/IgG/IgA F(ab’)2 fragments (dilution 1:250) for 30 minutes on ice. The BD Accuri C6 plus flow cytometer and FlowJo software (BD Biosciences, Becton, Dickinson and Company, Franklin Lakes, NJ, USA) were used to detect and analyze the expression of cell surface molecules on breast cancer cells.

### 2.7. Assessment of HuM6-1B9 Mediated ADCC

Target cells were labeled with 0.5 μM of 5,6-carboxyfluorescein diacetate succinimidyl ester (CFSE) (Sigma-Aldrich, Merck KGaA, Darmstadt, Germany) before being incubated with 10 µg/mL of HuM6-1B9 or trastuzumab or human IgG control for 30 min at room temperature. For co-culturing, PBMCs (effector cells) and antibody-coated target cells (1 × 10^4^ cells) were added in the FAC tube at an 80:1 effector-to-target (E:T) ratio and incubated at 37 °C for 4 h. Following incubation, 5 μg/mL of propidium iodide (PI) (Invitrogen, Thermo Fisher Scientific Inc., Waltham, MA, USA) was used to stain the dead cells. The CFSE^+^PI^+^ cells were identified as dead target cells.

To study the involvement of Fc gamma receptors (FcγRs) in ADCC, anti-FcγR antibodies were applied to inhibit ADCC. Effector cells were incubated with 10 μg/mL of mouse anti-FcγRIII (CD16) antibody (clone 3G8), anti-FcγRII (CD32) antibody (clone AT10), anti-FcγRI (CD64) antibody (clone 10.1), or mouse IgG1 isotype control (clone P3.6.2.8.1) for 30 min at 37 °C before co-culturing with the CFSE-labeled target cells. The percent of cytotoxicity was calculated as [(% of dead target cells—% of spontaneous death)/(100—% of spontaneous death)] × 100%.

### 2.8. Statistical Analysis

Data were analyzed using one-way analysis of variance (ANOVA) to compare the means of more than two groups. If a significant difference was detected, post hoc tests were performed to determine which specific groups differed from each other. All statistical analyses were conducted using GraphPad Prism 10 (GraphPad Software, San Diego, CA, USA).

## 3. Results

### 3.1. Assessment of Purity and Binding Activity of HuM6-1B9

**Figure 1:**
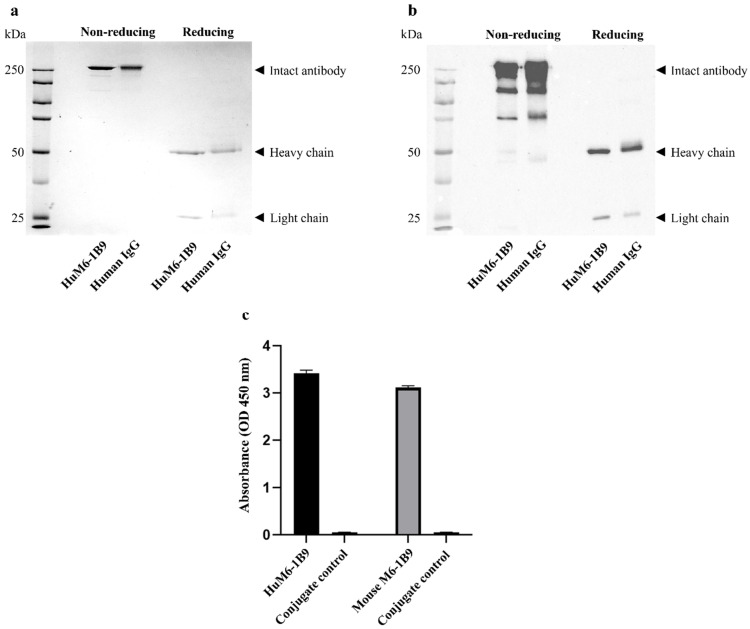
Characterization of the HuM6-1B9 antibody: (**a**) The purity of HuM6-1B9 is assessed by SDS-PAGE, followed by Coomassie blue staining. (**b**) The purified HuM6-1B9 on a nitrocellulose membrane is detected using rabbit anti-human IgG (H + L) antibody-HRP in a Western blot analysis. (**c**) The binding activity of HuM6-1B9 is evaluated using indirect ELISA (black), compared with mouse M6-1B9 (gray). The results are presented as a representative experiment from a total of three independent experiments.

To determine the purity and structure of HuM6-1B9, the purified protein was analyzed using SDS-PAGE under both non-reducing and reducing conditions. SDS-PAGE revealed a band above 150 kDa under non-reducing conditions, indicating the intact antibody structure. Under reducing condition, the results showed two distinct bands at approximately 50 kDa and 25 kDa, corresponding to the heavy and light chains of the antibody, respectively (Figure 1a). In Western blot analysis, these bands were detected with rabbit anti-human IgG (H + L) antibody-HRP, confirming the identity of HuM6-1B9 as a human IgG antibody (Figure 1b). Human IgG was included as a positive control and displaying an identical banding pattern under both non-reducing and reducing conditions. The size exclusion chromatography (SEC) analysis of HuM6-1B9 showed a major peak at 153.95 kDa, corresponding to intact IgG, and another at 88.77 kDa, with no detectable aggregation (Figure S1). Additionally, the binding activity of the purified HuM6-1B9 was demonstrated through indirect ELISA. HuM6-1B9 showed specific binding to CD147 coated on a 96-well plate (Figure 1c). The half-maximal effective concentration (EC_50_) value was determined to be 28.56 ng/mL (Figure S2).

### 3.2. Detection of CD147 and HER2 Molecules on Triple-Negative Breast Cancer Cells

**Figure 2:**
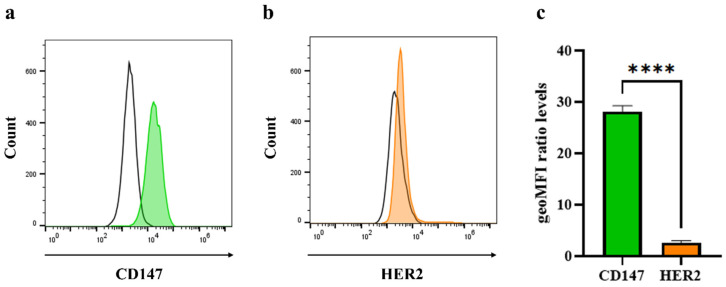
Expression of CD147 and HER2 on MDA-MB-231 cells: The cell surface molecules on MDA-MB-231 cells are determined using flow cytometry. The cells are incubated with HuM6-1B9 (green peak) (**a**) or trastuzumab (orange peak) (**b**) and stained with PE-labeled goat anti-human IgM/G/A F(ab’)2 fragments. The conjugate control is represented by the black line. Data of CD147 and HER2 expression represent one experiment from three independent experiments. (**c**) Fold change in the geo-MFI of CD147 and HER2 expression relative to the conjugate control is shown as mean ± SEM. Data are presented from three independent experiments. *p* values are calculated using unpaired *t* test, **** *p* < 0.0001.

CD147 expression levels on MDA-MB-231 cells were measured using cell surface staining. The histogram displaying CD147 expression is shown in Figure 2a. To confirm the characteristic of TNBC, which exhibits a lack of HER2 expression, the cells were analyzed using indirect immunofluoresce staining. The results demonstrated low HER2 expression on the cell surface (Figure 2b). The geometric mean fluorescence intensity (geo-MFI) of CD147 and HER2 was presented as a ratio relative to the conjugate control (Figure 2c), revealing that CD147 expression was significantly higher than HER2.

### 3.3. Evaluation of HuM6-1B9-Mediated ADCC in MDA-MB-231 Cells

**Figure 3:**
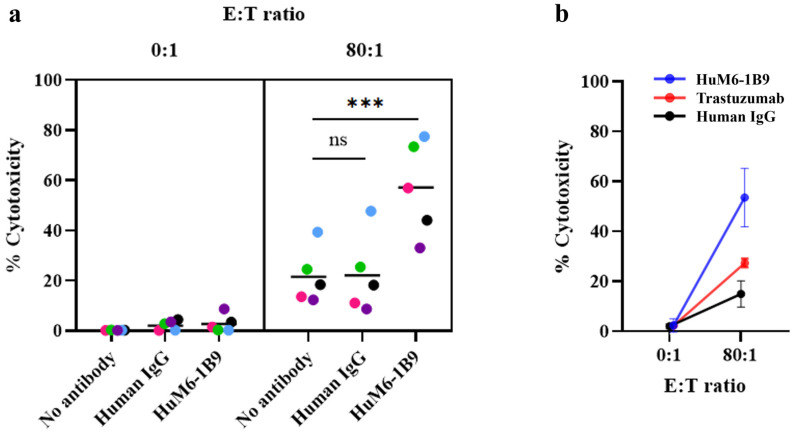
Investigation of the ADCC activity of the HuM6-1B9: CFSE-labeled MDA-MB-231 target cells are co-cultured with PBMCs, and cytotoxicity is assessed using PI staining. (**a**) The percentage of cytotoxicity of target cells is assessed by flow cytometry. Each color represents effector cells from five individual healthy donors. *p* values are calculated using one-way ANOVA followed by Tukey’s multiple comparison test, *** *p* < 0.0002. (**b**) Comparison of ADCC activity between HuM6-1B9 and trastuzumab in killing MDA-MB-231 cells. Mean values ± SEM are shown (n = 3).

As CD147 is overexpressed in TNBC, it could serve as an alternative therapeutic target for antibody-based therapy. Therefore, the efficacy of HuM6-1B9 on ADCC was investigated. MDA-MB-231 cells were labeled with CFSE and incubated with HuM6-1B9 to allow binding to the target antigen. After co-culturing with PBMCs (effector cells) for 4 h, cytotoxicity was assessed by flow cytometry (Figure 3a). In the absence of effector cells (E:T = 0:1), HuM6-1B9 alone did not induce significant cytotoxicity compared to the human IgG control, suggesting that HuM6-1B9 has no direct cytotoxic effect on MDA-MB-231 cells. Under co-culturing conditions at an E:T ratio of 80:1, the presence of HuM6-1B9 significantly enhanced the killing of target cells by the effector cells from five individual donors (mean ± SEM = 57.13 ± 8.47%), which was approximately three times higher than the cytotoxicity observed with the human IgG control (22.16 ± 7.04%). In the absence of HuM6-1B9, the percentage of target cell cytotoxicity was 21.58 ± 4.94%. Additionally, the efficacy of HuM6-1B9 in enhancing the ADCC of MDA-MB-231 at an E:T ratio of 80:1 was compared to trastuzumab using effector cells from three individual donors. The percentage of cytotoxicity in the presence of HuM6-1B9, trastuzumab, and the human IgG control in MDA-MB-231 cells were 54.01 ± 11.72%, 26.14 ± 1.71%, and 14.97 ± 5.22%, respectively (Figure 3b). These results suggest that HuM6-1B9 has greater potential in mediating TNBC cell death through ADCC compared to trastuzumab.

### 3.4. Identification of FcγR Involved in HuM6-1B9-Mediated ADCC

**Figure 4:**
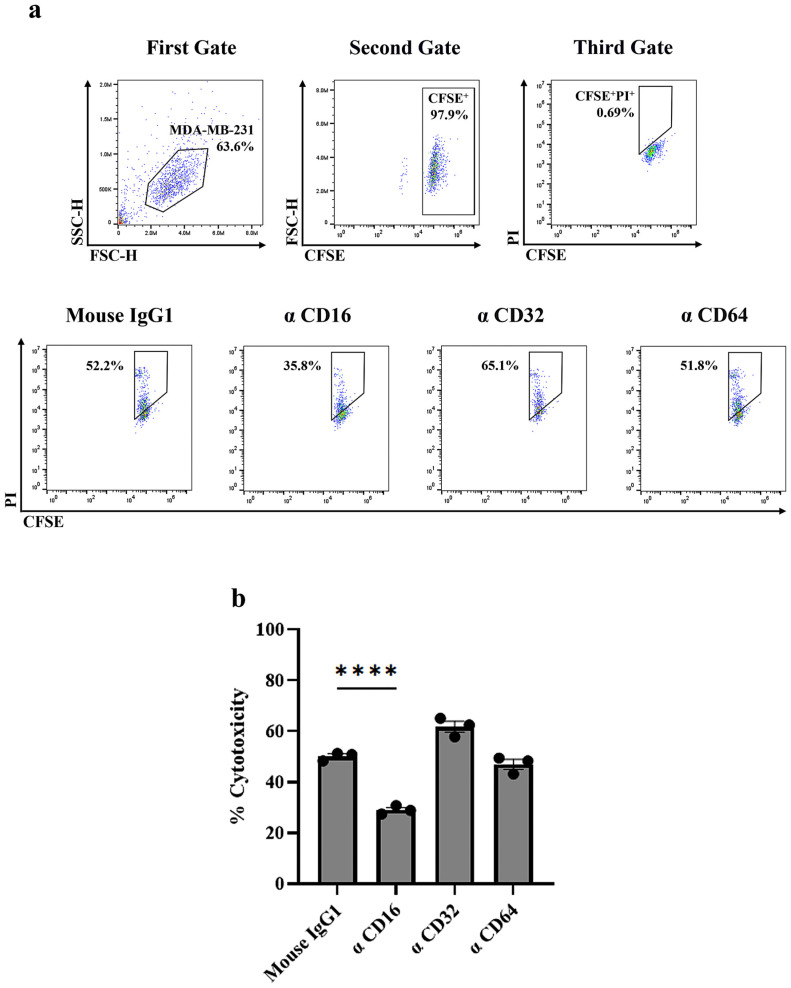
Determination of FcγR associated with HuM6-1B9-mediated ADCC. Before co-culturing with HuM6-1B9-sensitized, CFSE-labeled MDA-MB-231 target cells, effector cells are pre-incubated with anti-FcγR antibodies. (**a**) Gating strategy of the ADCC assay is shown in the first row. Target cell death induced by ADCC is analyzed by flow cytometry and represented as double-positive cells (CFSE⁺/PI⁺). The results shown are representative of one experiment out of three independent experiments, using PBMCs isolated from healthy donors. (**b**) ADCC inhibition assay using anti-FcγR monoclonal antibodies to block FcγR-mediated activity. The bar graph shows the percentage of cytotoxicity (mean ± SEM) from triplicate experiments. Statistical analysis is performed using one-way ANOVA with Tukey’s post hoc test. **** indicates *p* ≤ 0.0001.

To identify which FcγR mediates the ADCC activity of HuM6-1B9, the interaction between the Fc region of HuM6-1B9 and specific FcγR on effector cells was disrupted using anti-FcγR antibodies. The results showed that blocking FcγRIII (CD16) with an anti-CD16 antibody significantly reduced cytotoxicity (Figure 4). In contrast, blocking FcγRII (CD32) or FcγRI (CD64) had no significant effect on cytotoxicity. These findings highlight the critical role of FcγRIII in the ADCC activity of HuM6-1B9.

### 3.5. Investigation of HuM6-1B9 ADCC Activity Against PBMCs from Healthy Donors

**Figure 5:**
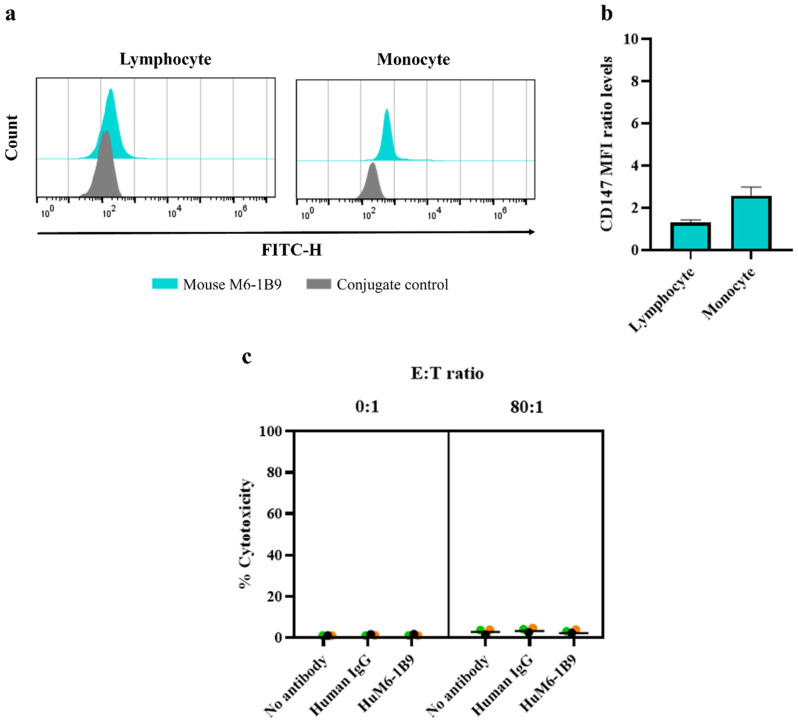
Examination of HuM6-1B9-mediated ADCC activity in PBMCs: (**a**) PBMCs are incubated with mouse M6-1B9 (blue peak), followed by staining with FITC-conjugated goat anti-mouse IgM/G antibodies. The white peak is a representation of conjugate control. The expression of CD147 on PBMCs is analyzed using flow cytometry. The results represent one of three healthy donors. (**b**) The relative geo-MFI of CD147 expression on PBMCs, including lymphocytes and monocytes, is demonstrated as mean ± SEM (n = 3). (**c**) PBMCs are used both as target and effector cells to assess whether HuM6-1B9 enhances self-killing mediated by the ADCC process. Each color represents effector cells from an individual healthy donor (n = 3).

The CD147 expression levels on PBMCs, including lymphocytes and monocytes, were analyzed using flow cytometry. The results showed low levels of CD147 expression on PBMCs (Figure 5a). The geo-MFI for lymphocytes and monocytes (mean ± SEM) was 145.00 ± 14.57 and 508.70 ± 68.28, respectively, compared to the geo-MFI of the conjugate control, which was 112.00 ± 0.00 and 196.70 ± 3.18. The ratio of the geo-MFI of CD147 expression was shown in Figure 5b. To further investigate whether HuM6-1B9 could induce off-target cytotoxicity, CFSE-labeled PBMCs were used as target cells in an ADCC assay. The target cells were incubated with HuM6-1B9, both in the absence and presence of effector cells at an 80:1 ratio, and the percentage of cytotoxicity was assessed. The results demonstrated that HuM6-1B9 did not promote the significant killing of PBMCs under any of the tested conditions (Figure 5c).

## 4. Discussion

Among breast cancer subtypes, TNBC is one of the most aggressive forms, with limited therapeutic options. Due to the lack of HER2 expression, patients cannot be treated with the humanized anti-HER2 monoclonal antibody, trastuzumab [30]. Several studies are attempting to identify alternative therapeutic markers for the development of antibody-based therapies. Novel targets, such as CD73 and CD146, have been studied in TNBC antibody therapy. CD73 is an ecto-5′-nucleotidase that converts AMP to adenosine, an immunosuppressive molecule that inhibits tumor-killing immune cells and is highly expressed in TNBC. Targeting CD73 with specific antibodies inhibits cell migration and invasion [31], while the anti-CD73 antibody–drug conjugate (ADC) anti-CD73-PLG-MMAE shows elicited potent cytotoxicity and suppresses tumor growth [32]. Although CD73 contributes to the tumor microenvironment by generating immunosuppressive adenosine, its direct influence on tumor cell survival and metastasis is less pronounced. CD146, which induces epithelial-to-mesenchymal transition (EMT) in cancer metastasis and contributes to the generation of cancer stem cells (CSCs), has emerged as another interesting target. Antibodies targeting soluble CD146, such as M2J-1 mAb, have demonstrated efficacy in counteracting its effects on proliferation, migration, and invasion in vitro, as well as on CSC and EMT markers both in vitro and in vivo [33]. Nevertheless, CD146 expression varies significantly across TNBC cases, limiting its universal applicability [34].

CD147 emerges as a particularly promising target, alongside CD73 and CD146, due to its significant overexpression in various malignancies, including TNBC [20,35]. CD147 plays crucial roles in tumor progression, invasion, and metastasis [35,36]. An anti-CD147 nanobody conjugated with doxorubicin has demonstrated antitumor activity in vitro and in vivo by inhibiting tumor cell proliferation and inducing apoptosis [37]. The advantage of nanobodies lies in their small size, which allows them to penetrate solid tumors. However, they lack the Fc region essential for immune-mediated functions [38]. Although the efficacy of nanobodies is enhanced by conjugating them with doxorubicin, which triggers apoptosis, high CD147 expression has been reported to be associated with doxorubicin resistance [36]. Therefore, inducing cytotoxicity with full-length anti-CD147 antibodies could be an effective strategy for improving the treatment of CD147-overexpressing cancer cells.

This study evaluates the anti-cancer activity, focusing on ADCC, of the humanized anti-CD147 monoclonal antibody HuM6-1B9 against MDA-MB-231 cells, a TNBC model. HuM6-1B9 demonstrated strong in vitro binding potency to CD147, with an EC_50_ of 28 ng/mL measured by ELISA. Our findings reveal that HuM6-1B9 mediated cancer cell killing by ADCC. Additionally, the ADCC mechanism depends on FcγRIII, as demonstrated by blocking experiments using an anti-CD16 antibody. This mechanism aligns with prior studies; for example, avelumab, a PD-L1–specific antibody, induces tumor killing by ADCC through FcγRIII [39,40]. The efficacy of HuM6-1B9 against MDA-MB-231 cells was evaluated in comparison to trastuzumab. The results demonstrated that HuM6-1B9 induces stronger ADCC activity than trastuzumab. This enhanced activity aligns with the differential expression levels of CD147 and HER2 in MDA-MB-231 cells, where CD147 is highly expressed, and HER2 expression is low. These findings suggest that HuM6-1B9 holds significant potential for the ADCC-mediated killing of breast cancer cells with elevated CD147 expression.

The major effector cells contributing to ADCC are NK cells, which mediate cytotoxicity against cancer cells through activating receptors and an antibody-dependent mechanism. Besides NK cells, CD16+ monocytes have been reported to contribute to ADCC by secreting pro-inflammatory cytokines such as TNF-α upon activation [41]. Our findings reveal that, in co-culture conditions without HuM6-1B9, the percentage of dead target cells was 21.58 ± 4.94%. This effect is likely mediated by NK cells within the PBMCs, which may recognize stress-induced ligands on tumor cells through activating receptors such as NKG2D [42]. Importantly, the presence of HuM6-1B9 significantly enhanced cancer cell killing by ADCC to 57.13 ± 8.47% and does not directly induce cytotoxicity in MDA-MB-231 cells. However, the potential cytotoxicity of HuM6-1B9 against normal cells remains a concern. To address this, we evaluated the expression of CD147 and the effect of HuM6-1B9 on PBMCs from healthy donors. The results showed that HuM6-1B9 did not induce cytotoxicity in PBMCs, likely due to the insufficient density of CD147 molecules to trigger ADCC. This finding supports the potential of HuM6-1B9 as a therapeutic antibody for cancer treatment. However, additional purification steps, such as ion exchange and SEC, are required to meet clinical study standards.

The variability in ADCC efficacy among patients is influenced by factors such as effector cell abundance, FcγRIIIa density, and *FCGR3A* gene polymorphisms (e.g., the low-affinity FcRIIIA-158F allele) [43,44]. Antibody engineering strategies, including Fc glycoengineering (e.g., afucosylation at Asn297) and amino acid mutations to enhance FcγRIIIa binding, have been shown to improve ADCC activity, as demonstrated by margetuximab [45,46,47,48]. To improve HuM6-1B9 efficacy in ADCC, Fc engineering is a feasible strategy. Furthermore, other immune-mediated functions, including CDC and ADCP, should be explored. These investigations will provide a more comprehensive understanding of HuM6-1B9-mediated immune function and therapeutic utility in TNBC.

The tumor microenvironment (TME) presents challenges to ADCC. For instance, approximately 76.6% of TNBC patients express PD-L1, an immune checkpoint protein that binds to PD-1 on immune cells such as T cells and NK cells, inhibiting their activation and reducing ADCC activity [49]. This highlights the potential benefit of combining antibody therapy with immune checkpoint inhibitors, such as pembrolizumab or nivolumab, to improve treatment efficacy. Alternatively, myeloid-derived suppressor cells (MDSCs) can produce immunosuppressive cytokines such as TGF-β and IL-10, resulting in the inhibition of NK cells. Our previous study reported that the knockout of CD147 in THP-1 cells suppresses their differentiation into monocyte-derived MDSCs [25]. These data suggest the crucial role of CD147 in impairing MDSC development. The utilization of CD16+ monocytes lacking CD147 as cell-based therapy, combined with antibody therapy, might be an alternative approach for TNBC treatment.

In some cases, TNBC can emerge from HER2-positive tumors due to tumor evolution or after anti-HER2 antibody treatment, often leading to HER2-negative diagnoses by immunohistochemistry (IHC) [50,51,52]. IHC has limitations in distinguishing HER2 = 0 from HER2-low, which can result in inappropriate treatment selection and an increased risk of recurrence. The Oncotype DX breast recurrence score test, which quantifies 21 genes including the HER2 gene using qRT-PCR, provides a more precise method to identify HER2-low expression, allowing for treatment with trastuzumab-deruxtecan (T-DXd) in patients, including some with TNBC [53,54]. Despite its efficacy, T-DXd’s high cost (USD 165,000/year) significantly limits accessibility, as it is 2.6 times more expensive than trastuzumab (USD 63,592/year) and 4.3 times higher than its biosimilar (USD 38,173/year) [55,56]. An alternative strategy for HER2-low metastatic breast cancer with CD147 upregulation is combination therapy with trastuzumab and HuM6-1B9, which could provide a synergistic ADCC effect to enhance cancer cell elimination at a more affordable cost.

Additionally, the dual-antibody approach may produce synergistic direct effects. The trastuzumab can disrupt cancer cell proliferation and survival by blocking HER2-mediated signaling pathways. At the same time, HuM6-1B9 may directly interfere with CD147-driven PI3K/Akt signaling, a key pathway involved in drug resistance [57,58], angiogenesis [59], and metastasis [60] in TNBC. Notably, PI3K/Akt inhibitors such as BKM120 and GDC-0980 have demonstrated potential in reducing cell proliferation and inducing apoptosis in TNBC cell lines [61,62]. Therefore, further studies should investigate the impact of HuM6-1B9 on the PI3K/Akt pathway, particularly its potential to inhibit angiogenesis and overcome resistance mechanisms in HER2-low metastatic breast cancer. This dual-antibody therapy could serve as an effective and accessible alternative for patients with HER2-low metastatic breast cancer, particularly those with CD147 upregulation in TNBC.

In conclusion, this study demonstrates that the humanized anti-CD147 antibody, HuM6-1B9, significantly enhances tumor cell killing through ADCC in MDA-MB-231 cells, a model for TNBC. HuM6-1B9 exhibits strong potential as a targeted therapy for TNBC and other cancers with CD147 overexpression, underscoring its promise for clinical translation. Notably, the antibody did not induce cytotoxicity in PBMCs, likely due to the insufficient density of CD147 molecules required to trigger ADCC, highlighting its favorable safety profile. These findings position HuM6-1B9 as a promising therapeutic candidate for TNBC, particularly given its superior efficacy compared to existing treatments like trastuzumab, which is ineffective in HER2-negative TNBC.

## Data Availability

The data presented in this study are available on request from the corresponding author upon reasonable request.

## Supplementary Materials

The following supporting information can be downloaded at https://www.mdpi.com/article/10.3390/antib14020036/s1, Figure S1: Size-exclusion chromatogram of HuM6-1B9. The protein-G purified HuM6-1B9 (250 µg) was analysed using a Superdex 200 Increase 10/300 GL column at a flow rate of 0.50 mL/min. Elution was carried out with 20 mM phosphate buffer (pH 7.0) containing 150 mM NaCl. The fractions, including the intact IgG and low molecular weight fraction (LMWF), were monitored by absorbance at 210 nm. The SEC standard curve is shown in the inset; Figure S2: The half maximal effective concentration (EC50) value of HuM6-1B9 binding to CD147 was determined using indirect ELISA. HuM6-1B9 (blue) was added in serial three-fold dilutions starting from 1 µg/mL, while human IgG (black) was used as an irrelevant control. The EC50 value was calculated using nonlinear regression analysis in GraphPad Prism 10. Data are presented as the mean ± S.D. from triplicate experiments.
