# Supplementary material for: Potentiating Antibody-Dependent Cellular Cytotoxicity in Triple-Negative Breast Cancer via the Humanized Anti-CD147 Antibody
Source: Antibodies (Basel). 2025 Apr 11;14(2):36. doi: 10.3390/antib14020036 (PMC12015854; doi:10.3390/antib14020036)
Supplement: Supplementary file 1 [file antibodies-14-00036-s001.zip › antibodies-3530038-supplementary.pdf]

Supplementary Materials

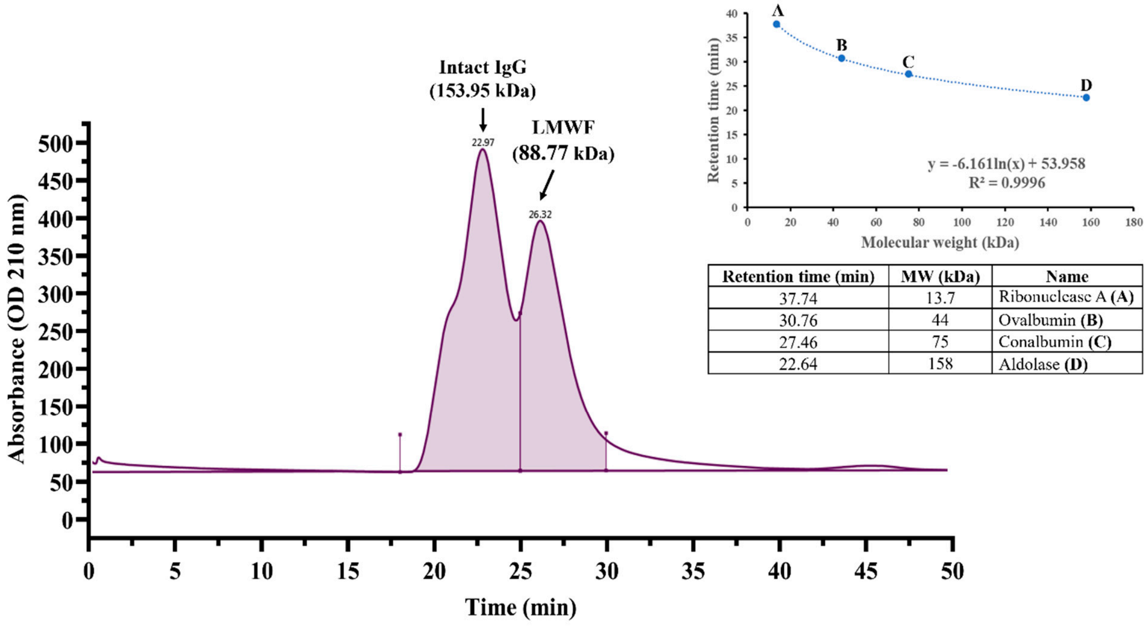

Retention time (min)	MW (kDa)	Name
37.74	13.7	Ribonuclease A **(A)**
30.76	44	Ovalbumin **(B)**
27.46	75	Conalbumin **(C)**
22.64	158	Aldolase **(D)**

Figure S1. Size-exclusion chromatogram of HuM6-1B9. The protein-G purified HuM6-1B9 (250 µg) was analysed using a Superdex 200 Increase 10/300 GL column at a flow rate of 0.50 mL/min. Elution was carried out with 20 mM phosphate buffer (pH 7.0) containing 150 mM NaCl. The fractions, including the intact IgG and low molecular weight fraction (LMWF), were monitored by absorbance at 210 nm. The SEC standard curve is shown in the inset.

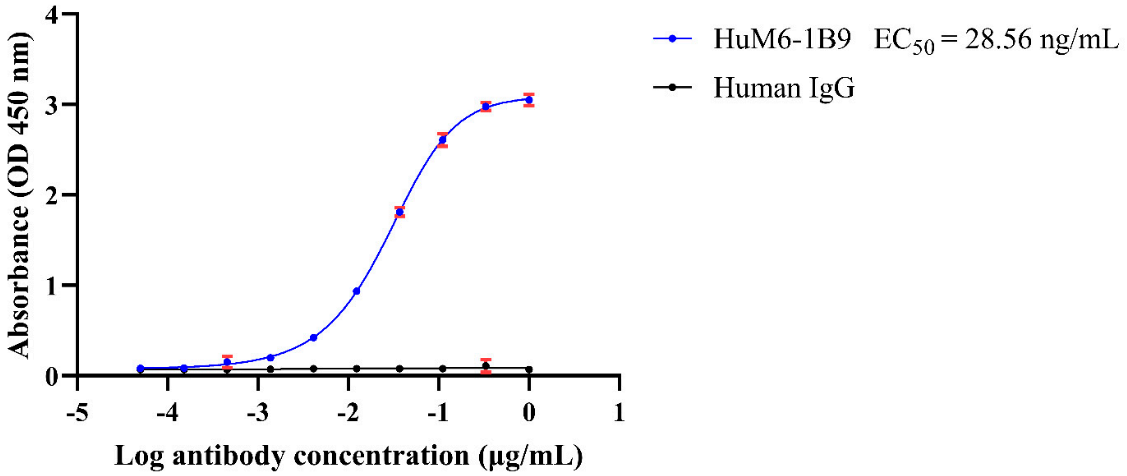

Figure S2. The half maximal effective concentration (EC_{50}) value of HuM6-1B9 binding to CD147 was determined using indirect ELISA. HuM6-1B9 (blue) was added in serial three-fold dilutions starting from 1 µg/mL, while human IgG (black) was used as an irrelevant control. The EC_{50} value was calculated using nonlinear regression analysis in GraphPad Prism 10. Data are presented as the mean ± S.D. from triplicate experiments.
